# Assessment of Pre-operative Factors Associated With Blood Loss in Patients Undergoing Percutaneous Nephrolithotomy: A Prospective Study

**DOI:** 10.7759/cureus.53739

**Published:** 2024-02-06

**Authors:** Deelip K Singh, Sanjay Gupta, Kumar Shubham, Nandesh Kumar, Rajesh Tiwari

**Affiliations:** 1 Urology, Indira Gandhi Institute of Medical Sciences, Patna, IND; 2 General Surgery, All India Institute of Medical Sciences, Patna, Patna, IND

**Keywords:** urological surgery, factors causing blood loss, blood loss in pcnl, percutaneous nephrolithotomy, pcnl

## Abstract

Background

One of the main risks associated with percutaneous nephrolithotomy (PCNL) is bleeding. In the present study, efforts are made to evaluate the pre-operative predictive factors contributing to bleeding due to the procedure of PCNL.

Materials and methods

From December 2019 to November 2021, data were collected prospectively from 193 patients undergoing PCNL procedures at Indira Gandhi Institute of Medical Sciences, Patna, India. Following PCNL, to check for hematuria and the extent of blood loss, the urethral catheter's and nephrostomy tube's outputs were evaluated. Multivariate regression analysis was used to evaluate the relationship between blood loss and a variety of patient-related demographic and clinical characteristics.

Results

Included in the study were 193 patients who underwent PCNL. Male patients made up the majority. The average age of study participants was 33.5 years. No statistically significant difference was reported in the mean hemoglobin level drop in the age groups of up to 25 years (2.211 ± 1.540 g/dL), 26-50 years (2.023 ± 1.882 g/dL), and > 50 years (1.855 ± 0.986 g/dL) with P = 0.64. The mean hemoglobin level drop in patients with stone burden > 30 mm^2^ was reported to be higher, 2.359 ± 1.822 g/dL, compared to 1.859 ± 1.540 g/dL in patients with lower stone burden, reaching a statistically significant difference (P =0.0408). By univariate regression analysis, the presence of a horseshoe-shaped kidney (odds ratio = -0.158, 95% confidence interval (CI): -0.911, -0.059; P = 0.026) was associated with a higher risk for a drop in mean hemoglobin level. By multivariate regression analysis, the presence of a horseshoe-shaped kidney (odds ratio = 0.071, 95% CI: 0.006, 0.839; P = 0.036) remained significantly and independently associated with a higher risk of a drop in mean hemoglobin level.

Conclusion

In conclusion, the patients' burden of stones and the presence of a horseshoe-shaped kidney may be associated with a higher risk of bleeding following PCNL.

## Introduction

Percutaneous nephrolithotomy (PCNL) is the standard of care for large renal calculi and has largely surpassed open surgical techniques in renal stone management [1]. The primary goal of treatment is the absolute clearance of stones with minimal complications.

Despite recent advances, complications related to PCNL are still common. A noteworthy study by the Clinical Research Office of the Endourological Society group reported complications in about one-fifth of the patients undergoing PCNL [2]. Complications of PCNL include fever, infection, and post-operative complications, such as infundibular stenosis. Bleeding is the most common and significant complication of PCNL, with the reported incidence ranging from 7% to 20% [3]. Arterial damage with the subsequent development of arteriovenous fistulas or pseudoaneurysms is a well-known source of bleeding after PCNL [4]. Other causes of bleeding may be damage to the intercostal artery or venous injury in the renal parenchyma [5].

Patients with a comparatively high risk of bleeding following the procedure may benefit from secondary prevention efforts, given the high frequency and variable course of nephrolithiasis. However, our present understanding of the demographic and clinical factors linked to a greater likelihood of bleeding after a PCNL procedure is lacking. In the present study, efforts are made to evaluate the pre-operative predictive factors contributing to bleeding in patients undergoing PCNL.

## Materials and methods

Study population

The present study prospectively reviewed the data of patients with renal stones who underwent PCNL at the Indira Gandhi Institute of Medical Sciences, Patna, India, from December 2019 to November 2021. Out of these, we identified patients who had significant postoperative bleeding. Criteria for significant bleeding used in our study included hemoglobin level drop > 2 g/dL, the requirement of blood transfusion, the requirement of intervention in the form of angioembolization, or hemodynamic instability (increasing pallor, hypotension, cardiorespiratory distress).

Prospectively identified patients of either sex, aged 18 years or older with renal stones, who were admitted to the urology department for the procedure of PCNL, were included. Patients with infected hydronephrosis or pyonephrosis, renal insufficiency, pregnancy, uncorrected coagulopathy, or urinary tract infection were excluded from this study.

Study procedure and data collection

Departmental Research Committee and Institutional Ethics Committee approval (approval number: 1201/IEC/IGIMS/2019) was obtained prior to the commencement of the study. All the patients were given a patient information sheet that provided a complete description of the study prior to patient enrollment. Written and informed consent was obtained from all patients. Several preoperative factors were assessed, which included age, sex, pre-existing comorbidities, history of previous intervention, blood group, stone burden, hardness of stone, location of stone, type of stone, and degree of hydronephrosis.

Preoperatively, a hemogram, serum creatinine, serum electrolytes, coagulation profile, blood sugar levels, urine culture, and ultrasound of the kidneys, ureters, and bladder were performed in all patients. Patients with normal serum creatinine underwent a urography with computed tomography (CT urography), while those with elevated serum creatinine levels (>1.4 mg/dL) underwent a non-contract CT of the abdomen, followed by a functional renal scan.

PCNL technique: Under general anesthesia in the lithotomy position, a cystoscopy was done, and a 5F ureteric catheter was inserted. In the prone position, puncture was done using an 18 G two-piece needle in sites depending on the location of the stone in the prone position with a metallic trocar and a cannula of size 12 F. Dilatation was done using a fascial dilator of size 28 or 30 F. Lithotripsy was done using a pneumatic lithotripter. Picking up large fragments was the next step, followed by the insertion of a Double-J stent (DJ stent) or ureteric catheter in situ and the placement of a nephrostomy tube. Stone clearance was documented with X-rays of the kidney, ureter, and bladder (X-ray KUB).

Until six hours postoperatively, patients were kept nil orally with intravenous fluid support. Vital signs such as temperature, pulse rate, blood pressure, and urine output were monitored. Postoperative hematocrit and intraoperative and immediate postoperative outputs from the nephrostomy tube and the urethral catheter were assessed to check for hematuria and the degree of blood loss. During the six to 24 hours of the postoperative period, oral intake was allowed, vital signs were monitored, and blood investigations such as a complete blood count and kidney function test were done. The following schedule in Table 1 was followed for the patients.

**Table 1:** Assessments and interventions done in the post-operative period.

Post-operative Day (POD)	Assessments and Interventions
POD 1	Routine vitals monitoring, chest physiotherapy, and initiation of a normal diet.
POD 2	Chest X-ray, X-ray KUB, and ureteric catheter (if placed) removal.
POD 3	Nephrostomy tube removal (if placed).
POD 4	Urethral catheter removal.
POD 5	The patient was discharged if there was no hematuria, fever, or nephrostomy site soakage.

Patients were called for DJ stent removal after two to three weeks, and then regular follow-up every three months was done for one year.

In cases of post-PCNL bleeding, patients with mild and moderate hematuria were respectively managed by a conservative or blood transfusion approach. Three patients presented with delayed bleeding a few days after discharge but within three weeks of surgery. These patients were stabilized with intravenous fluids and blood transfusions.

Statistical analysis

Descriptive statistics were analyzed in Microsoft Excel software (Microsoft Corporation, Washington, DC). Descriptive statistics were used to describe demographic and clinical characteristics. All other hypotheses and analyses were performed using Statistical Product and Service Solutions (SPSS, version 20; IBM SPSS Statistics for Windows, Armonk, NY) statistical software. A chi-square or Fisher's exact test was done to find out the association between categorical variables. The Spearman correlation coefficient was used to estimate the correlation between two continuous variables. A P value of less than 0.05 was considered significant.

## Results

A total of 193 patients who had undergone PCNL were included in this study. The average age of the patients was 33.5 years. Patients were grouped into three groups according to age: up to 25 years old, 26-50 years old, and more than 50 years old. No statistically significant difference was reported in the mean hemoglobin level drop among three different age groups (Table 2).

**Table 2:** Mean hemoglobin level drop in patients of various age groups.

Age groups	Number of patients	Mean hemoglobin level drop in g/dL	P value
Up to 25 years	69	2.211 ± 1.540	P = 0.6436
26-50 years	104	2.023 ± 1.882	
>50 years	20	1.855 ± 0.986	

The mean hemoglobin level drop in male patients (n = 117, 60%) was 2.229 ± 1.966 g/dL, compared to 1.833 ± 1.104 g/dL in female patients (n = 76, 40%), with no statistically significant difference (P = 0.11). With respect to pre-existing comorbidities, seven patients (3.6%) had hypertension, five patients (2.6%) had type 2 diabetes mellitus, and one patient had hypothyroidism. Horseshoe-shaped kidney was reported in four patients (2.1%), and polio-kyphoscoliosis was reported in one patient. A history of previous interventions was reported in four patients (nephrolithotomy, coronary artery bypass surgery, PCNL, and aortic valve surgery). Blood group O was the most common, comprising 33.2% of patients, followed by blood group A (28.5%) and blood group B (25.9%). Blood group AB was the least common, at 10.9%. Out of all the patients, 99.5% were Rh positive, and the rest (0.5%) were Rh negative. The average hardness of stone measured in terms of Hounsfield units (HU) was 1,255.1 HU. The mean hemoglobin level drop in patients with stone hardness in the range of 500-1,000 HU was 1.540 ± 1.771 g/dL, as compared to 2.153 ± 1.678 g/dL in patients with stone hardness in the range of 1,000-1,500 HU. However, the difference was not statistically significant (P = 0.126).

The average stone burden was 34.3 mm^2^. The hemoglobin level drop reported in patients with stone burden > 30 mm^2^ was 2.359 ± 1.822 g/dL, compared to 1.859 ± 1.540 g/dL in patients with lower stone burden, showing a statistically significant difference (P = 0.0408). The renal pelvis accounted for 73.58% of cases of nephrolithiasis, followed by the lower calyx (33.16%), upper calyx (7.77%), and middle calyx (4.15%). However, there was no significant difference in hemoglobin level drop based on the location of the stone. Stone analysis showed that 174 patients (90.16%) had calcium oxalate stones, 11 patients (5.7%) had uric acid stones, and only eight patients (4.15%) had mixed stones. No statistically significant difference was reported in the hemoglobin level drop in relation to the type of stone (Table 3).

**Table 3:** Mean hemoglobin level drop in patients with different types of stone.

Type of stone	Number of patients	Hemoglobin level drop in g/dL	P value
Calcium oxalate	174	2.117 ± 1.676	P = 0.38
Uric acid	11	2.088 ± 0.035	
Mixed stones	8	1.364 ± 2.350	

The majority of the patients (n = 97, 50.3%) showed mild hydronephrosis, while 31.6% and 2.6% of patients had moderate and severe hydronephrosis, respectively. However, no statistically significant difference in hemoglobin level drop was reported with respect to the degree of hydronephrosis (Table 4).

**Table 4:** Mean hemoglobin level drop in patients with different degrees of hydronephrosis.

Degree of hydronephrosis	Number of patients	Mean hemoglobin level drop in g/dL	P value
Mild	97	2.222 ± 0.190	P = 0.5292
Moderate	61	1.969 ± 0.193	
Severe	5	2.700 ± 1.177	

By univariate regression analysis, the presence of a horseshoe-shaped kidney (odds ratio = -0.158, 95% CI: -0.911, -0.059; P = 0.026) was associated with a higher risk for a drop in mean hemoglobin level. By multivariate regression analysis, the presence of a horseshoe-shaped kidney (odds ratio = 0.071, 95% CI: 0.006, 0.839; P = 0.036) remained significantly and independently associated with a higher risk of a drop in mean hemoglobin level.

## Discussion

The incidence and prevalence of kidney stone disease are increasing globally. It affects all ages but occurs more frequently within the age range of 20-49 years [6]. While flexible ureteroscopic stone removal and extracorporeal shock wave lithotripsy (ESWL) are common treatment techniques for renal stones, PCNL is still required in certain circumstances depending on the size, composition, position, and shape of the stone [7]. However, compared to flexible ureteroscopic stone removal, or ESWL, the PCNL procedure is much more invasive. Hence, it comes with a considerable morbidity risk. While the majority of PCNL-related bleeding may be controlled conservatively, about 0.8% of patients need intervention to stop severe bleeding [8]. Determining the variables linked to elevated bleeding risk may facilitate a broader application of bleeding prevention following PCNL. With the use of this information, medical professionals may be able to concentrate their preventive efforts on individuals who are more likely to bleed and who stand to gain significantly from such an intervention. We evaluated the associations between several clinical and demographic characteristics of the patients who underwent PCNL for nephrolithiasis and the risk of bleeding due to the intervention.

A study by Said et al. on 200 patients, reported no significant effect of age, sex, body mass index, diabetes, or hypertension on blood loss [9]. However, the presence of intraoperative pelvicalyceal perforation, history of ipsilateral nephrolithiasis surgery, and stone complexity were concerning variables for post-PCNL hemorrhage. Kukreja et al. did not report any association of age with bleeding [10]. In our study, a total of 193 patients who had undergone PCNL were included. The average age of the patients was 33.5 years, and we sub-categorized the patients into three categories and found no statistically significant difference in the mean hemoglobin level drop. Similarly, men were more than women, with a men-to-women ratio of 1.5:1, and the difference in mean hemoglobin level drop was not statistically significant.

Previous research has indicated that stone size is a significant predictor of bleeding after PCNL. Meng et al., Lee et al., and Kukreja et al. reported stone size to be a risk factor for bleeding following PCNL [10-12]. In our study, the mean hemoglobin level drop in stone burden > 30 mm^2^ was reported to be significantly greater as compared to stone burden <30 mm^2^. Similarly, two other studies have reported stag-horn stones, diabetes, and stone size as predictive variables for bleeding during PCNL [13,14]. The presence of horseshoe-shaped kidneys was also reported to be a significant risk factor for bleeding in our study.

There are a few limitations to our study. It is single-center research with short follow-up. We were unable to conduct a meaningful analysis of the impacts of race and ethnicity due to the high levels of homogeneity in terms of race and ethnicity in our sample. Due to these constraints and the requirement for validation of our results, we are unable to offer any recommendations regarding the care of patients whose estimated risk of bleeding following PCNL differs. However, the current study's findings imply that the patient's burden of stones and the presence of a horseshoe-shaped kidney may be associated with a higher risk of bleeding during PCNL.

## Conclusions

Based on the results of a single institutional study, we conclude that individuals with a significant stone burden and the presence of horseshoe-shaped kidneys, but not age, gender, pre-existing diabetes, hypertension, type of stone, or hardness of stone, have a higher risk of bleeding from the procedure of PCNL. Before doing PCNL, endourologists should consider if patients have the above-mentioned risk factors since prevention is more important than treatment. Regular reporting of preoperative hemoglobin levels is necessary for the analysis of PCNL outcome studies.
